# Supplementary material for: SKAP2 is required for defense against K. pneumoniae infection and neutrophil respiratory burst
Source: eLife. 2020 Apr 30;9:e56656. doi: 10.7554/eLife.56656 (PMC7250567; doi:10.7554/eLife.56656)
Supplement: Supplementary file 1. — WT and Skap2-/- (KO) DIV neutrophils were infected with wild-type Kp, or stimulated with IgG IC (as positive control) for 10 min at 37°C. Lysates were prepared and analyzed by western blot for phosphoproteins, total proteins, and RhoGDI as indicated. Quantification of protein level was assessed using Licor. [file elife-56656-supp1.docx]

**Supplemental Table 1**

|  | Normalized phospho-SFK intensity^a^ | | | | | | | |
| --- | --- | --- | --- | --- | --- | --- | --- | --- |
|  | WT | | | KO | | | WT | KO |
|  | Uns | *Kp* 10' | *Kp* 15' | Uns | *Kp* 10' | *Kp* 15' | IgG IC 10' | IgG IC 10' |
| Experiment 1 | 3.27 | 4.85 | 8.64 | 2.80 | 3.87 | 4.21 | 8.16 | 6.18 |
| Experiment 2 | 1.15 | 2.33 | 5.18 | 1.02 | 1.39 | 2.49 | 3.63 | 2.23 |
| Experiment 3 | 1.03 | 2.67 | 3.14 | 1.52 | 2.39 | 1.99 | 3.03 | 3.07 |
|  | Fold change phospho-SFKs to uns within each group^b^ | | | | | | | |
|  | WT | | | KO | | | WT | KO |
|  | Uns^c^ | *Kp* 10' | *Kp* 15' | Uns | *Kp* 10' | *Kp* 15' | IgG IC 10' | IgG IC 10' |
| Experiment 1 | 1.00 (1.80) | 1.48 | 2.64 | 1.00 | 1.38 | 1.50 | 2.49 | 2.21 |
| Experiment 2 | 1.00 (0.63) | 2.03 | 4.52 | 1.00 | 1.37 | 2.45 | 3.17 | 2.19 |
| Experiment 3 | 1.00 (0.57) | 2.58 | 3.03 | 1.00 | 1.57 | 1.31 | 2.93 | 2.02 |
|  | Fold change phospho-SFKs to BALB/c Uns^d^ | | | | | | | |
|  | WT | | | KO | | | WT | KO |
|  | Uns | *Kp* 10' | *Kp* 15' | Uns | *Kp* 10' | *Kp* 15' | IgG IC 10' | IgG IC 10' |
| Experiment 1 | 1.00 | 1.48 | 2.64 | 0.85 | 1.18 | 1.29 | 2.49 | 1.89 |
| Experiment 2 | 1.00 | 2.03 | 4.52 | 0.89 | 1.22 | 2.17 | 3.17 | 1.94 |
| Experiment 3 | 1.00 | 2.58 | 3.03 | 1.47 | 2.31 | 1.92 | 2.93 | 2.97 |
| WT and *SKAP-2-/-* (KO) DIV neutrophils were infected with wild-type Kp, or stimulated with IgG IC (as positive control) for 10 minutes at 37ºC. Lysates were prepared and analyzed by western blot for phospho-SFKs, and RhoGDI (loading control). Blots were then stripped and re-probed for the respective total protein. Quantification of protein level was assessed using Licor. ^a^ Normalized phospho-SFKs = ratio of the intensity of phosphorylated bands to RhoGDI loading control. ^b^ Fold change was calculated by diving the normalized values of stimulated samples by the normalized value of unstimulated (uns) sample within respective group of each experiment.  ^c^ Fold change in parenthesis was calculated by diving the normalized values of unstimulated samples of each experiment by the average normalized value of unstimulated (uns) sample from all three experiments. These values are the ones shown in Fig. 5D. ^d^ Fold change was calculated by diving the normalized values of stimulated samples by the normalized value of WT unstimulated (uns) sample of each experiment. | | | | | | | | |

**Supplemental Table 2**

|  | Normalized phospho-Syk intensity^a^ | | | | | | | |
| --- | --- | --- | --- | --- | --- | --- | --- | --- |
|  | WT | | | KO | | | WT | KO |
|  | Uns | *Kp* 10' | *Kp* 15' | Uns | *Kp* 10' | *Kp* 15' | IgG IC 10' | IgG IC 10' |
| Experiment 1 | 5.47 | 16.09 | 9.97 | 4.51 | 9.36 | 6.69 | 30.24 | 28.85 |
| Experiment 2 | 7.04 | 40.04 | 8.50 | 6.67 | 11.50 | 7.40 | 29.84 | 23.80 |
| Experiment 3 | 3.29 | 11.36 | 9.56 | 4.77 | 8.65 | 4.60 | 32.03 | 28.27 |
|  | Fold change of phospho-Syk to uns within each group^b^ | | | | | | | |
|  | WT | | | KO | | | WT | KO |
|  | Uns^c^ | *Kp* 10' | *Kp* 15' | Uns | *Kp* 10' | *Kp* 15' | IgG IC 10' | IgG IC 10' |
| Experiment 1 | 1.00 (1.25) | 2.94 | 1.82 | 1.00 | 2.07 | 1.48 | 5.53 | 6.39 |
| Experiment 2 | 1.00 (1.47) | 5.69 | 1.21 | 1.00 | 1.72 | 1.11 | 4.24 | 3.57 |
| Experiment 3 | 1.00 (0.30) | 3.46 | 2.91 | 1.00 | 1.81 | 0.97 | 9.74 | 5.93 |
|  | Fold change of phospho-Syk to BALB/c Uns^d^ | | | | | | | |
|  | WT | | | KO | | | WT | KO |
|  | Uns | *Kp* 10' | *Kp* 15' | Uns | *Kp* 10' | *Kp* 15' | IgG IC 10' | IgG IC 10' |
| Experiment 1 | 1.00 | 2.94 | 1.82 | 0.83 | 1.71 | 1.22 | 5.53 | 5.28 |
| Experiment 2 | 1.00 | 5.69 | 1.21 | 0.95 | 1.63 | 1.05 | 4.24 | 3.38 |
| Experiment 3 | 1.00 | 3.46 | 2.91 | 1.45 | 2.63 | 1.40 | 9.74 | 8.60 |
| WT and *SKAP-2-/-* (KO) DIV neutrophils were infected with wild-type Kp, or stimulated with IgG IC (as positive control) for 10 minutes at 37ºC. Lysates were prepared and analyzed by western blot for phospho-Syk Y352 and RhoGDI (loading control). Blots were then stripped and re-probed for the respective total protein. Quantification of protein level was assessed using Licor. ^a^ Normalized phospho-Syk = ratio of the intensity of phosphorylated bands to total protein to RhoGDI loading control. ^b^ Fold change was calculated by diving the normalized values of stimulated samples by the normalized value of unstimulated (uns) sample within respective group of each experiment.  ^c^ Fold change in parenthesis was calculated by diving the normalized values of unstimulated samples of each experiment by the average normalized value of unstimulated (uns) sample from all three experiments. These values are the ones shown in Fig. 5F. ^d^ Fold change was calculated by diving the normalized values of stimulated samples by the normalized value of WT unstimulated (uns) sample of each experiment. | | | | | | | | |

**Supplemental Table 3**

|  | Normalized phospho-Pyk2 intensity^a^ | | | | | | | |
| --- | --- | --- | --- | --- | --- | --- | --- | --- |
|  | WT | | | KO | | | WT | KO |
|  | Uns | *Kp* 10' | *Kp* 15' | Uns | *Kp* 10' | *Kp* 15' | IgG IC 10' | IgG IC 10' |
| Experiment 1 | 26.32 | 122.05 | 101.24 | 33.10 | 57.20 | 38.05 | 198.06 | 239.76 |
| Experiment 2 | 5.46 | 42.88 | 66.21 | 10.86 | 21.37 | 21.08 | 96.51 | 92.52 |
| Experiment 3 | 8.36 | 40.49 | 32.77 | 15.50 | 21.20 | 17.73 | 39.91 | 27.07 |
|  | Fold change of phospho-Pyk2 to uns within each group^b^ | | | | | | | |
|  | WT | | | KO | | | WT | KO |
|  | Uns^c^ | *Kp* 10' | *Kp* 15' | Uns | *Kp* 10' | *Kp* 15' | IgG IC 10' | IgG IC 10' |
| Experiment 1 | 1.00 (2.34) | 4.64 | 3.85 | 1.00 | 1.73 | 1.15 | 7.53 | 7.24 |
| Experiment 2 | 1.00 (0.30) | 7.85 | 12.13 | 1.00 | 1.97 | 1.94 | 17.68 | 8.52 |
| Experiment 3 | 1.00 (0.37) | 4.84 | 3.92 | 1.00 | 1.37 | 1.14 | 4.77 | 1.75 |
|  | Fold change of phospho-Pyk2 to BALB/c Uns^d^ | | | | | | | |
|  | WT | | | KO | | | WT | KO |
|  | Uns | *Kp* 10' | *Kp* 15' | Uns | *Kp* 10' | *Kp* 15' | IgG IC 10' | IgG IC 10' |
| Experiment 1 | 1.00 | 4.64 | 3.85 | 1.26 | 2.17 | 1.45 | 7.53 | 9.11 |
| Experiment 2 | 1.00 | 7.85 | 12.13 | 1.99 | 3.91 | 3.86 | 17.68 | 16.95 |
| Experiment 3 | 1.00 | 4.84 | 3.92 | 1.85 | 2.54 | 2.12 | 4.77 | 3.24 |
| WT and *SKAP-2-/-* (KO) DIV neutrophils were infected with wild-type Kp, or stimulated with IgG IC (as positive control) for 10 minutes at 37ºC. Lysates were prepared and analyzed by western blot for phospho-Pyk2 and RhoGDI (loading control). Blots were then stripped and re-probed for the respective total protein. Quantification of protein level was assessed using Licor. ^a^ Normalized phospho-Pyk2 = ratio of the intensity of phosphorylated bands to total protein to RhoGDI loading control. ^b^ Fold change was calculated by diving the normalized values of stimulated samples by the normalized value of unstimulated (uns) sample within respective group of each experiment.  ^c^ Fold change in parenthesis was calculated by diving the normalized values of unstimulated samples of each experiment by the average normalized value of unstimulated (uns) sample from all three experiments. These values are the ones shown in Fig. 5F. ^d^ Fold change was calculated by diving the normalized values of stimulated samples by the normalized value of WT unstimulated (uns) sample of each experiment. | | | | | | | | |
