# Supplementary material for: SKAP2 is required for defense against K. pneumoniae infection and neutrophil respiratory burst
Source: eLife. 2020 Apr 30;9:e56656. doi: 10.7554/eLife.56656 (PMC7250567; doi:10.7554/eLife.56656)
Supplement: Transparent reporting form [file elife-56656-transrepform.pdf]

eLIFE
1st Floor
24 Hills Road
Cambridge CB2 1JP, UK
P 01223 855340
W elifesciences.org
T @elife

eLife's transparent reporting form

We encourage authors to provide detailed information *within their submission* to facilitate the interpretation and replication of experiments. Authors can upload supporting documentation to indicate the use of appropriate reporting guidelines for health-related research (see EQUATOR Network), life science research (see the BioSharing Information Resource), or the ARRIVE guidelines for reporting work involving animal research. Where applicable, authors should refer to any relevant reporting standards documents in this form.

If you have any questions, please consult our Journal Policies and/or contact us: editorial@elifesciences.org.

Sample-size estimation

- You should state whether an appropriate sample size was computed when the study was being designed
- You should state the statistical method of sample size computation and any required assumptions
- If no explicit power analysis was used, you should describe how you decided what sample (replicate) size (number) to use

Please outline where this information can be found within the submission (e.g., sections or figure legends), or explain why this information doesn't apply to your submission:

Previous work with lung infection of gram-negative bacteria have shown that we could detect a significant difference in 10 fold levels in CFU with 5 mice with 95% confidence and 4 fold levels in CFU with 7 mice. Therefore, we estimated that we would need between 5-8 mice per group.

Replicates

- You should report how often each experiment was performed
- You should include a definition of biological versus technical replication
- The data obtained should be provided and sufficient information should be provided to indicate the number of independent biological and/or technical replicates
- If you encountered any outliers, you should describe how these were handled
- Criteria for exclusion/inclusion of data should be clearly stated
- High-throughput sequence data should be uploaded before submission, with a private link for reviewers provided (these are available from both GEO and ArrayExpress)

Please outline where this information can be found within the submission (e.g., sections or figure legends), or explain why this information doesn't apply to your submission:

eLife Sciences Publications, Ltd is a limited liability non-profit non-stock corporation incorporated in the State of Delaware, USA, with company number 5030732, and is registered in the UK with company number FC030576 and branch number BR015634 at the address 1st Floor, 24 Hills Road, Cambridge CB2 1JP | August 2014

The number of replicates and statistical tests for each test are reported within the figure legends. For cell culture-based assays with differentiated in vitro (DIV) HoxB8 cells or bone marrow derived neutrophils, biological replicates are where we performed the assays on different days. So for biological replicates, bone marrow neutrophils are harvested from different mice on different days and DIV neutrophils were differentiated on different days. In addition, we have used DIV neutrophils derived from different mice in many assays. Technical replicates are where we performed the same assay on the same sample multiple times in the same day. For the cell-based assays such as phagocytosis and reactive oxygen species production, experiments are performed in technical triplicate. Averages of technical triplicates were calculated and used in the calculation of means of the experiment and the summary statistics are based on three independent biological replicates and SEM, unless explicitly mentioned in the figure legends (i.e. any data that is a representative plot is reported as means $\pm$ SD of technical triplicates and used for statistics).

For Western blot analysis, experiments are done on at least three different days with different preparations of DIV neutrophils. Multiple proteins are assessed each experiment/day. Each protein's level/experiment is quantified and each is reported as a biological replicate.

For animal experiments, we consider each mouse as a biological replicate and typically we test 2-3 mice/day/condition. In addition, we test the same conditions on different cohorts of mice on different days and combine all the data. Therefore, all animal experiments are performed on 2-4 different days with 2-4 mice/time point/genotype unless otherwise indicated in the figure legends. All data is combined and analyzed as described in Figure legends and materials and methods. In all cases, the CFU values are log10 transformed.

Raw or normalized data are presented in graphs as individual points and means, averages, or geometric means are presented as lines or bars in the same graph.

eLife Sciences Publications, Ltd is a limited liability non-profit non-stock corporation incorporated in the State of Delaware, USA, with company number 5030732, and is registered in the UK with company number FC030576 and branch number BR015634 at the address 1st Floor, 24 Hills Road, Cambridge CB2 1JP | August 2014

Statistical reporting

- Statistical analysis methods should be described and justified
- Raw data should be presented in figures whenever informative to do so (typically when N per group is less than 10)
- For each experiment, you should identify the statistical tests used, exact values of N, definitions of center, methods of multiple test correction, and dispersion and precision measures (e.g., mean, median, SD, SEM, confidence intervals; and, for the major substantive results, a measure of effect size (e.g., Pearson's r, Cohen's d)
- Report exact p-values wherever possible alongside the summary statistics and 95% confidence intervals. These should be reported for all key questions and not only when the p-value is less than 0.05.

Please outline where this information can be found within the submission (e.g., sections or figure legends), or explain why this information doesn't apply to your submission:

Statistical analyses used are stated in the Methods and figure legends. Raw data are included in figures, and details of how they were generated are indicated in the methods and figure legends where appropriate.

(For large datasets, or papers with a very large number of statistical tests, you may upload a single table file with tests, Ns, etc., with reference to sections in the manuscript.)

Group allocation

- Indicate how samples were allocated into experimental groups (in the case of clinical studies, please specify allocation to treatment method); if randomization was used, please also state if restricted randomization was applied
- Indicate if masking was used during group allocation, data collection and/or data analysis

Please outline where this information can be found within the submission (e.g., sections or figure legends), or explain why this information doesn't apply to your submission:

Mice were originally housed in cages of 5 mice per cage and housed together for 2-12 days prior to experiment. Mice were then moved into a BSL-2 facility and randomly assigned to smaller group sizes for each cohort.
Masking was used during evaluation of HE staining – at least 4 people analyzed slides without knowing the experimental cohorts (or even what and how many experimental cohorts were being evaluated).

Additional data files ("source data")

- We encourage you to upload relevant additional data files, such as numerical data that are represented as a graph in a figure, or as a summary table
- Where provided, these should be in the most useful format, and they can be uploaded as "Source data" files linked to a main figure or table
- Include model definition files including the full list of parameters used
- Include code used for data analysis (e.g., R, MatLab)
- Avoid stating that data files are "available upon request"

Please indicate the figures or tables for which source data files have been provided:

Data including averages, means or geometric means of experiments are shown. No large data sets were generated in this work.

eLife Sciences Publications, Ltd is a limited liability non-profit non-stock corporation incorporated in the State of Delaware, USA, with company number 5030732, and is registered in the UK with company number FC030576 and branch number BR015634 at the address 1st Floor, 24 Hills Road, Cambridge CB2 1JP | August 2014
